# Rapid Buildup of Genetic Diversity in Founder Populations of the Gynodioecious Plant Species *Origanum vulgare* after Semi-Natural Grassland Restoration

**DOI:** 10.1371/journal.pone.0067255

**Published:** 2013-06-19

**Authors:** Kenny Helsen, Hans Jacquemyn, Martin Hermy, Katrien Vandepitte, Olivier Honnay

**Affiliations:** 1 Plant Conservation and Population Biology, Department of Biology, University of Leuven, Leuven, Belgium; 2 Division Forest, Nature and Landscape Research, Department of Earth and Environmental Sciences, University of Leuven, Leuven, Belgium; University of Gottingen, Germany

## Abstract

In most landscapes the success of habitat restoration is largely dependent on spontaneous colonization of plant species. This colonization process, and the outcome of restoration practices, can only be considered successful if the genetic makeup of founding populations is not eroded through founder effects and subsequent genetic drift. Here we used 10 microsatellite markers to investigate the genetic effects of recent colonization of the long-lived gynodioecious species *Origanum vulgare* in restored semi-natural grassland patches. We compared the genetic diversity and differentiation of fourteen recent populations with that of thirteen old, putative source populations, and we evaluated the effects of spatial configuration of the populations on colonization patterns. We did not observe decreased genetic diversity in recent populations, or inflated genetic differentiation among them. Nevertheless, a significantly higher inbreeding coefficient was observed in recent populations, although this was not associated with negative fitness effects. Overall population genetic differentiation was low (F_ST_ = 0.040). Individuals of restored populations were assigned to on average 6.1 different source populations (likely following the ‘migrant pool’ model). Gene flow was, however, affected by the spatial configuration of the grasslands, with gene flow into the recent populations mainly originating from nearby source populations. This study demonstrates how spontaneous colonization after habitat restoration can lead to viable populations in a relatively short time, overcoming pronounced founder effects, when several source populations are nearby. Restored populations can therefore rapidly act as stepping stones and sources of genetic diversity, likely increasing overall metapopulation viability of the study species.

## Introduction

In many landscapes, large scale habitat restoration has proven to be the only way to establish self-sustaining ecosystems that are resilient to future perturbation [1], [2]. The success of these restoration schemes always depends to some extent on spontaneous colonization of the newly created habitats by plant species. The long term viability of these newly established populations and even communities, however, can be expected to be heavily dependent on their genetic makeup [3]–[5]. Therefore, it is of high importance to get insight into the processes that affect genetic diversity of plant populations that have colonized restored habitats [6]. So far, there are only few reports on the genetic effects of spontaneous plant colonization directly following ecological restoration practices, e.g. [7]–[9].

Metapopulation genetic theory can help to understand the potentially complex genetic consequences of early colonization following habitat restoration [10]. Since colonization often involves the establishment of a limited number of founding individuals, only a subsample of the genetic variability of the source populations will be present in colonizing populations. These founder effects, or genetic bottlenecks, reduce local population genetic diversity and can result in large genetic differentiation between colonizing populations, especially when population growth rates remain small after colonization [11], [12]. In the same way, these bottlenecks can cause an increase in non-random associations between pairs of loci (linkage disequilibrium), which in turn may accelerate stochastic loss of genetic diversity when random genetic drift due to small initial population sizes persists in these founder populations [13], [14].

To what extent founder events reduce population genetic diversity and increase the magnitude of genetic differentiation among populations is dependent on (i) the number of colonizing individuals that arrive in the new habitat, relative to the number of migrating individuals between extant populations, and (ii) the degree of common source population origin of colonizing propagules [10], [15]–[18]. In general, genetic founder effects are predicted to be strong when population establishment is mediated by few colonists from a limited number of source populations (the ‘propagule pool’ model). Founder effects are predicted to be weak or even absent, on the other hand, when colonization occurs from multiple source populations (the ‘migrant pool’ model) [10], [19]. The probability (*φ*) that two colonizing individuals originate from the same source population (with *φ* = 1 for the propagule pool model and *φ* = 0 for the migrant pool model) is therefore a good indication of the extent of founder effects [16]. Increased genetic differentiation between founder populations is expected if the inequality 
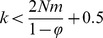
 holds, with *k* the number of colonists and *Nm* the effective number of migrants [16], [18].

As the extent of genetic founder effects is strongly mediated by the amount and the direction of gene flow between populations, it can be expected to depend strongly on the spatial configuration and the connectivity of the restored populations and the source populations [20]. Limited founder effects can be expected, for instance, when many source populations are present that are well connected to the recent populations, either by small geographical distance, a permeable landscape matrix, a high intrinsic potential for gene flow of the colonizing species, or a combination of any of these factors [21], [22]. Taking into account the position of possible source populations, relative to restored habitat patches, will therefore help to better understand the temporal and spatial patterns of genetic diversity in restored plant populations, possibly resulting in clearer guidelines for future ecological restoration schemes [23]–[25].

In this study we investigated the genetic consequences of recent colonization of the long-lived plant species *Origanum vulgare* (Lamiaceae) in newly restored semi-natural grassland patches. This species is a diploid (2*n* = 30), aromatic perennial herb, mainly occurring in grasslands on relatively dry, nutrient-poor to moderately nutrient-rich calcareous soils [26]. Individual plants can live up to 5–50 years and flowering can occur a year after germination, during summer and early fall [27], [28]. The species is self-compatible, but mainly outcrossing (facultative allogamous) due to protandry, and is pollinated by insects, mainly bees and bumblebees [27]. *Origanum vulgare* exhibits gynodioecy, in which both hermaphroditic and functionally female individuals co-occur [29], [30]. Female plants of *O. vulgare* bare exclusively male-sterile flowers, which contain one receptive stigma and four aborted (sterile) anthers and are considerably smaller than those of hermaphroditic plants [31]. The sex ratio of natural populations of *O. vulgare* in Western Europe has been found to vary between 1–62% of male sterility [32]. Propagation is accomplished by a large number of small seeds (mean weight 0.1 mg), which germinate in vegetation gaps during spring. Seed dispersal occurs through autochory, anemochory and epizoochory [27]. The seeds can form a persistent seed bank, with a seed bank longevity index (LI) of 0.41 *sensu* [33], indicating that *O. vulgare* was classified as forming a persistent seed bank in 41% of all performed seed bank studies including this species [27]. In only 23% of the studies observing persistent seeds for *O. vulgare*, seeds were found to survive longer than 5 years in the soil [27]. Vegetative reproduction also occurs through a rhizome-like pleiocorm.

To investigate the genetic consequences of the recent colonization of *Origanum vulgare*, we compared genetic diversity, linkage disequilibrium and genetic differentiation between fourteen recently colonized populations and thirteen old established populations, using 10 highly polymorphic microsatellite markers. Since we sampled a large number of possible source populations, we were able to investigate whether the spatial configuration of source populations, relative to the colonizing populations, mediated patterns of gene flow between old and recent populations, and influenced the degree of genetic differentiation among recent populations. Because our study system consisted of a large set of recently restored grassland patches, all founder populations were of relatively recent origin (<10 years old). This allowed us to examine the impact of source population genetic diversity on the genetic makeup of the recent populations, after only very limited effects of possible drift and inbreeding effects, which are expected to become more likely with increasing number of generations and thus with aging [34]. Many other studies have been unable to disentangle these effects, since the study system included older founder populations [35]–[37].

The sex ratio in natural populations of gynodioecious plant species can be highly variable, particularly in recently established populations, due to random sampling effects during colonization [38], [31]. Skewed sex ratios can have a major impact on patterns of genetic diversity, for example by promoting cross-fertilization and therefore enhancing population genetic diversity and reducing the severity of occurring founder effects or by limiting fertilization when recent populations are dominated by female plants, in turn enhancing the severity of occurring founder effects [39]–[42]. For this reason sex ratio was included as a possible explanatory variable of population genetic diversity and structure. More specifically we asked the following questions:

1. Does the spatial configuration of old relative to recent populations of *Origanum vulgare* structure genetic colonization patterns?
2. Are recent populations of *O. vulgare* characterized by impoverished genetic diversity, increased linkage disequilibrium and inflated genetic differentiation, caused by founder events?
3. Do our results concur with either the ‘propagule pool’ or the ‘migrant pool’ model of Slatkin [10]?

## Materials and Methods

### Study area

The study area comprises several calcareous grasslands in the valley of the river Viroin (Southern Belgium) and covers c. 80 km^2^ (c.50°N, 4.5°E). These mature grasslands occur on stony hills ranging in altitude from 150 to 250 m and are surrounded by forests and cultivated land [43]. During the 20^th^ century, most grasslands had become small and highly fragmented due to plantation of *Pinus sylvestris* and *P. nigra* and the abandonment of grazing, often leading to *Buxus sempervirens* encroachment. Recent restoration practices between 2001 and 2007 have led to the creation of ca. 100 ha restored historical calcareous grassland through the removal of trees and shrubs, which reduced the spatial isolation of the remaining grasslands [44]. In our study area, *O. vulgare* is mainly restricted to remnant and restored calcareous grasslands. After tree and shrub removal, spontaneous colonization of typical grassland species was allowed. Colonization of grassland species was only facilitated through the movement of sheep across grassland patches for grazing, with no deliberate introduction of seeds or seedlings. *Origanum vulgare* responded very quickly to restoration practices and was successful at establishing large populations on several recently restored grasslands.

### Sampling and laboratory procedures

**Figure 1:**
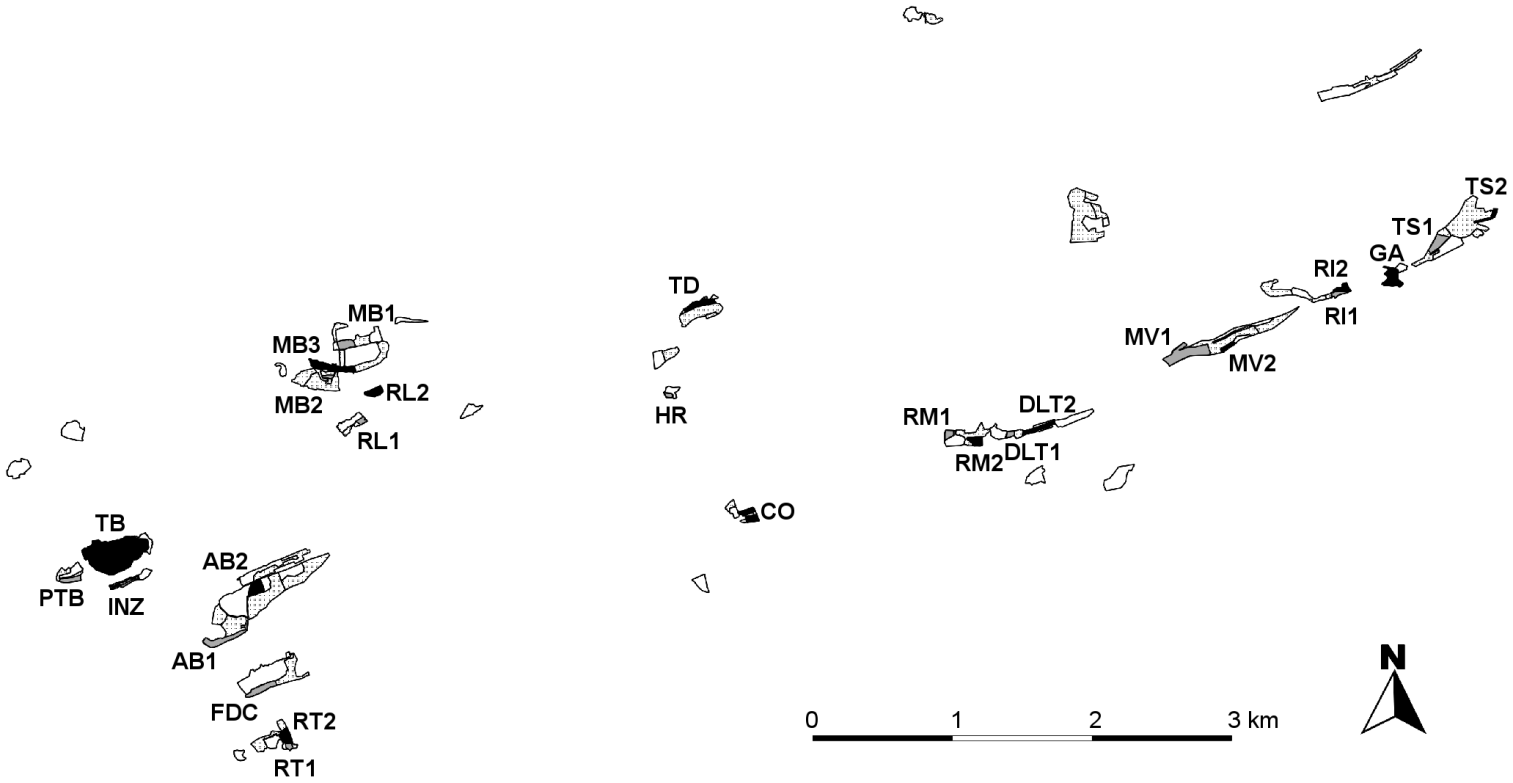
Study area in the Viroin valley. Figure visualises the sampled recent populations (grey) and old populations (black) of *O. vulgare*. Ancient (white) and restored (dotted) calcareous grasslands are visualised as well. Population codes correspond to those in table 1.

In total, we randomly selected 27 populations: 14 populations were located in recently restored calcareous grasslands, and 13 populations in mature calcareous grasslands (Fig. 1). In the summer of 2011, leaf material of 20 randomly selected individuals per population was collected and dried on silica gel. For each population, population size and the percentage of female plants were determined by counting the number of female and hermaphroditic individuals. For the largest populations (>1500 individuals) only a part of the population was counted and an approximation of the total populations size was extrapolated from this counted subset. Seed material of 25 plants per population was collected in October of 2011 and pooled per sampling location. All necessary permits were obtained for the described study, which complied with all relevant regulations. Permit for fieldwork in the Viroin-Hermeton national park (Parc naturel Viroin-Hermeton) was obtained through Léon Woué, by the CNB (Société royale - Cercles des Naturalistes de Belgique).

In the laboratory mean seed weight per population was obtained using a high precision balance (accuracy = 0.001 g). For each population, three replicates of 20 seeds were placed on moist Whatman paper in separate 10-mm Petri dishes. Seeds were incubated at 5°C for 2 weeks, followed by 4 weeks at 20°C with a 12h photoperiod. The number of germinated seeds was counted daily. Reproductive success per population was quantified using two estimates: mean seed weight and the percentage of germinated seeds (germination rate) at the end of the experiment.

We extracted DNA from collected leaf samples using the Nucleospin DNA-extraction kit (Macherey Nagel, Germany). DNA quality and concentration were estimated using a NanoDrop ND-2000 spectrophotometer (Thermo Scientific, Wilmington, DE, USA). For this study we used ten microsatellites developed by Novak et al. [45] (OR 10, 12-14, 27, 40, 44, 64, 75 & 77). Amplifications of the DNA were carried out in two multiplexes of five microsatellites using dyeset DS-33 (Applied Biosystems, CA, USA) in 10 µL reactions containing 1 µL template DNA, 2 µL of one of the two multiplexed primer combinations (both forward and reverse primers, 1 µM), 5 µL Qiagen Multiplex PCR Master Mix and 2 µL RNAse-free water. The polymerase chain reaction (PCR) was performed using a 2720 Thermal Cycler (Applied Biosystems, CA, USA). The PCR cycling profile of Novak et al. [45] was used, starting with an initial denaturation at 95°C for 15 min, followed by 35 cycles of 1 min at 95°C, 1 min at 59°C and 2 min at 72°C, with a final extension step of 9 min at 72 °C. After PCR, 1 mL of reaction was added to a solution of 8.8 mL formamide and 0.2 mL of the Applied Biosystems’ GeneScan 500 LIZ size standard. Fragments were sized on an ABI Prism, 3130 Genetic Analyzer (Applied Biosystems) and scored with GeneMapper Software v4.0 (Applied Biosystems). Raw microsatellite data for all 27 populations are available from the Dryad Digital Repository: http://dx.doi.org/10.5061/dryad.08gc2.

### Data analysis

After checking the microsatellite data for scoring errors due to stutter bands, null alleles and large allele dropout with MICRO-CHECKER [46], the mean number of alleles per population (A), expected heterozygosity (H_E_) and observed heterozygosity (H_O_) were calculated for each population using GenAlEx 6.5 [47]. The inbreeding coefficient (F_IS_) was estimated based on Wright`s F-statistics with GenAlEx 6.5 [47]. We tested for the occurrence of composite linkage disequilibrium between each pair of loci in each population with exact probability tests in Genepop 4.0.10 [48]. This test applies Markov chain algorithms on all contingency tables corresponding to all possible pairs of loci within each population. For each population, we summed the number of allele pairs for which significant linkage disequilibrium occurred. We used this metric as an indication of severity of linkage disequilibrium (LD) for each population. We then tested for the occurrence of recent bottleneck events in each population by looking for evidence of excess heterozygosity relative to allele numbers using the Bottleneck software [49]. We used a two-phase model of mutation (TPM) with a 90% stepwise component, which is considered most appropriate for microsatellite data [49].

To test for the occurrence of founder effects on the different metrics of within population genetic diversity we used first-order factorial General Linear Models (GLM) in Statistica 10 [50] using A, H_O_, H_E_, F_IS_ and LD as dependent variables. The models contained population age (recent vs. old) as a factor, and population size and the percentage of female plants as covariates and all first order interaction terms. All non-significant terms were removed using stepwise model reduction to obtain the final models. Population size and the percentage of female plants (%F) were log transformed to obtain homogeneity of the variances. We tested for effects of population age and population size on the percentage of female flowers with an analogue GLM model. We also performed a Levene’s test to test for differences in variance in the percentage of female flowers between recent and old populations. Mean population size was compared between restored and old populations with a t-test. To test for founder effects at the level of reproductive success, mean seed weight and germination rate were correlated to F_IS_ using Pearson correlations and compared between recent and old populations using a t-test.

Pairwise genetic differentiation among populations based on Wright`s F-statistics (F_ST_) was calculated. Because genetic differentiation measured by F_ST_ may be underestimated for multi-allelic markers, such as microsatellites [51], we also calculated Hedrick’s G’_ST_ and Jost’s D which are not affected by marker variability. G’_ST_ is the original G_ST_ as defined by Nei [52] standardized by the maximum value it can obtain (G_ST(max)_) [53]. Jost’s D is calculated based on the effective number of alleles instead of heterozygosity, which is considered a more intuitive diversity estimate [54]. All three pairwise genetic differentiation metrics were calculated and their significance was inferred based on 9999 permutations in GenAlEx 6.5 [47].

Genetic differentiation among populations was compared between recent and old populations based on a 2-tailed t-test on the pairwise F_ST,_ G’_ST_ and Jost’s D values. Because of the dependence of pairwise data, a bootstrapping procedure of 9999 bootstraps was applied for the calculation of the test statistics and the mean values and 95% confidence intervals for recent and old populations separately (SPSS Statistics 19.0). An analogous analysis was performed to compare pairwise geographical distances between recent and old populations. Total genetic diversity was partitioned among recent and old populations (among groups), among populations and within populations by performing a hierarchical analysis of molecular variance (AMOVA) on F_ST_ with GenAlEx 6.5 [47]. Significance of these genetic differentiations was tested based on 9999 permutations. Isolation by distance was tested for all populations and for recent and old populations separately, by regressing pairwise genetic (F_ST_, G’_ST_ and Jost’s D) distances on pairwise logarithmic spatial distances using Mantel tests in GenAlEx 6.5 [47]. A total of 9999 random permutations were performed. Geographical distances between populations were calculated as the Euclidean distance between population centroids using QGIS 1.7.4 (Quantum GIS Development Team 2010).

To characterize the overall genetic structure, we applied a Bayesian clustering approach implemented in STRUCTURE 2.3.3 [55]. We used the admixture model with correlated alleles applying burn-ins of 10^4^ and runs of 10^5^ repetitions for each value of *K* (number of population clusters), varying from 1 to 16. We performed 20 iterations for each tested value of *K*. The entire model was also rerun using the LOCPRIOR option. This allows the model to use population identity of the individuals as prior information to assist the clustering. The true number of *K* for both models was identified based on the approach of Evanno et al. [56].

Finally, to infer colonization patterns, individual plants of recent populations were assigned to old populations based on the Monte Carlo resampling procedure [57] implemented in GeneClass2 [58]. This procedure makes use of the allele frequency distributions, using a Bayesian approach to assign individuals, with assignment probabilities based on a threshold of *P* = 0.05. Based on the assignment analysis, we calculated *φ_p_* for each recent population, i.e. the probability that two gene flow events into a recent population originate from the same source population, by combinations of probabilities [14]. The *φ_p_* for each recent population was calculated as 
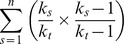
, with s the number of source populations of which plants originated in the recent population *p* (with *p* = 1 to *m*), *k_s_* the number of plants in the recent population *p* genetically originating from source population *s* (with *s* = 1 to *n*) and *k_t_* the total number of plants in the recent population (in this case the number of sampled individuals in each recent population, *k_t_* = 20). The overall value of *φ_p_* for the study system was calculated as the mean *φ_p_* over all recent populations. To infer the effects of spatial configuration on gene flow, we correlated the geographical distance between each recent population and the different source populations, with the number of plant individuals assigned to each of these source populations. Since these data points are not independent, we performed a linear regression with bootstrapping (10000 bootstraps) (SPSS Statistics 19.0).

## Results

### Genetic diversity

MICRO-CHECKER results indicated no problems with scoring errors due to stutters or allelic dropout in any of the 10 loci. We did, however, detect the occurrence of a homozygote excess in 22% of the populations for 2 loci (OR 12 and OR 75), possibly indicating the occurrence of null alleles. For this reason, all analyses were rerun excluding these loci. As the obtained results were similar to those obtained using all 10 loci, we decided to include all 10 loci in the analyses.

**Figure 2:**
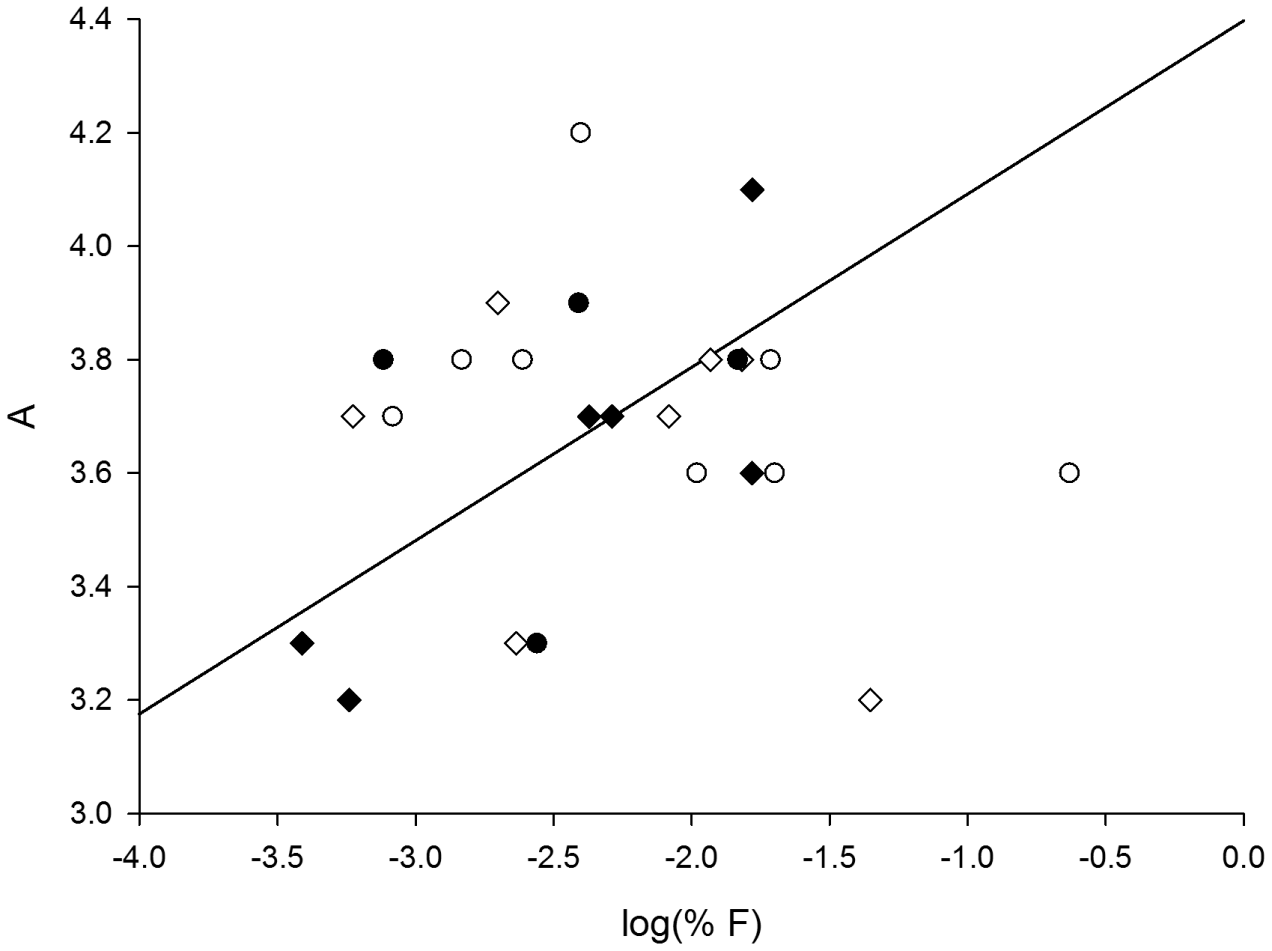
Relation between the mean number of alleles (A) and the percentage of female plants (%F). The relation is visualized independent for small (<500 plants, open symbol, no regression line shown) (*r* = –0.29, *P* = 0.29) and large populations (>1000 plants, full symbol, continuous line) (*r* = 0.64, *P* = 0.048). Recent populations are presented as diamonds, old populations as circles. %F was log transformed.

**Figure 3:**
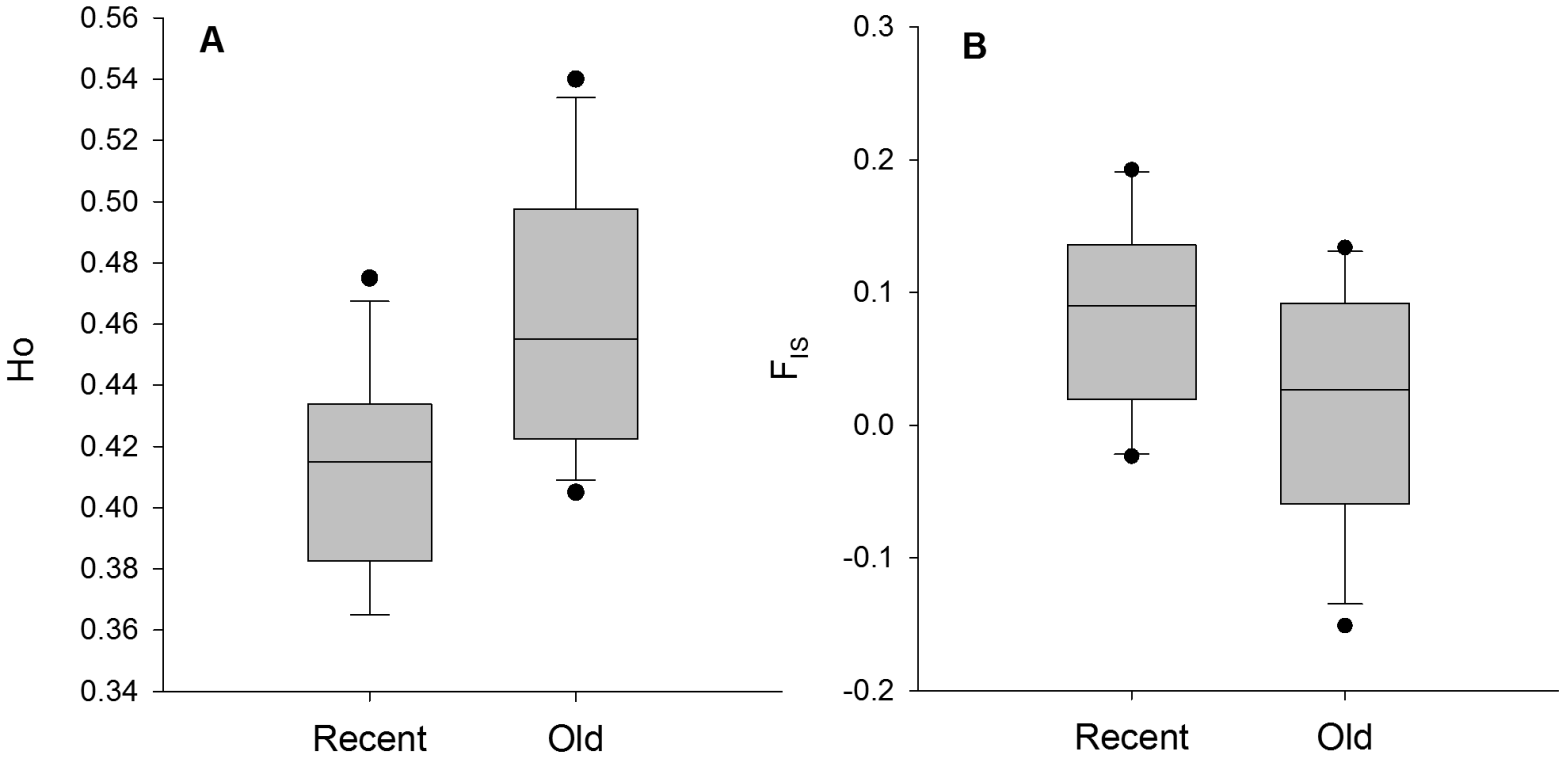
Difference in observed heterozygosity and inbreeding coefficient between recent and old populations. A. Boxplot for observed heterozygosity (H_O_). B. Boxplot for inbreeding coefficient (F_IS_).

Recent populations had a median of 708 plants (range: 150–5000), with median of 9.7% female plants (range: 3.3–25.9%). The number of alleles per population (A) varied between 3.1 and 4.1 alleles per population (average: 3.6), whereas observed heterozygosity varied between 0.37 and 0.48 (average: 0.42) for recent populations (Table 1). Old populations had a median of 9.1% female plants (range: 4.6–53.2%) with a median population size of 459 plants (range: 99–2250), a mean A of 3.7 (range: 3.3–4.2) and a mean H_O_ of 0.44 (range: 0.41–0.54) (Table 1). No significant difference between recent and old populations was found for population size (*t* = 1.0, *P* = 0.31), the percentage of female flowers (*t* = –2.2, *P* = 0.56) or the variance in the percentage of female flowers (*F* = 1.2, *P* = 0.76). The percentage of female flowers was not related to population size. The mean number of alleles increased with increasing percentage of female plants. However, this pattern was influenced by population size, with a decrease in the positive correlation with decreasing population size (significant interaction term, Table 2). This was visualized by dividing population size in small (<500 plants) and large populations (>1000 plants), and performing a Pearson correlation test independently for small and large populations (Fig. 2). Both H_O_ and F_IS_ were affected by population age, with a significantly lower observed heterozygosity and higher inbreeding in recent populations (Table 2, Fig. 3). Expected heterozygosity (H_E_) was not affected by any of the measured population characteristics. Linkage disequilibrium (LD) on the other hand, decreased with increasing population size, but was unaffected by population age (Table 2). When we tested for a correlation between LD and population size for recent and old populations independently, we found a significant correlation for old populations (*r* = –0.61, *P* = 0.027), but not for recent populations (*r* = –0.43, *P* = 0.12). We found no evidence of recent genetic bottlenecks in any of the 27 populations. Reproductive success was not affected by F_IS_ (germination rate: *r* = 0.025, *P* = 0.90; seed weight: *r* = –0.084, *P* = 0.68) or population age (germination rate: *t* = –1.068, *P* = 0.30; seed weight: *t* = 0.26, *P* = 0.80).

**Table 1:** Population characteristics of all sampled *Origanum vulgare* populations for recent and old populations separately.

Recent population	pop. size	% F	A	H_E_	H_O_	F_IS_	LD
AB1	3000	16.9	3.6	0.45	0.39	0.13	1
DLT1	348	12.5	3.7	0.49	0.48	0.058	0
FDC	2000	3.3	3.3	0.47	0.37	0.19	1
HR	923	8.0	3.1	0.40	0.37	0.014	1
INZ	298	14.5	3.8	0.45	0.43	0.021	0
MB1	1088	9.3	3.7	0.48	0.43	0.12	2
MB2	250	16.3	3.8	0.51	0.43	0.11	2
MV1	5000	16.9	4.1	0.48	0.39	0.19	0
PTB	491	4.0	3.7	0.46	0.38	0.14	3
RI1	150	25.9	3.2	0.42	0.40	0.036	5
RL1	1250	10.1	3.7	0.46	0.46	–0.021	2
RM1	198	6.7	3.9	0.46	0.43	0.069	2
RT1	492	7.2	3.3	0.49	0.41	0.14	2
TS1	2250	3.9	3.2	0.45	0.45	–0.023	2
**Mean (±SD)**	**1267 (±1386.9)**	**11.1 (±6.4)**	**3.6 (±0.30)**	**0.46 (±0.029)**	**0.42 (±0.034)**	**0.084 (±0.072)**	**1.6 (±1.3)**
**Old population**	**pop. size**	**% F**	**A**	**H_E_**	**H_O_**	**F_IS_**	**LD**
AB2	261	7.3	3.8	0.45	0.51	–0.11	1
CO	2250	9.0	3.9	0.53	0.53	0.021	1
DLT2	701	13.7	3.6	0.47	0.54	–0.15	3
GA	1500	16.0	3.8	0.47	0.49	–0.040	1
MB3	478	9.1	4.2	0.52	0.44	0.13	2
MV2	282	18.3	3.6	0.48	0.43	0.042	3
RI2	443	13.8	3.6	0.50	0.46	0.092	2
RL2	1325	4.4	3.8	0.48	0.45	0.051	0
RM2	1300	7.7	3.3	0.43	0.42	–0.014	3
RT2	110	5.9	3.8	0.51	0.49	0.027	6
TB	99	53.2	3.6	0.42	0.46	–0.079	2
TD	147	4.6	3.7	0.46	0.42	0.092	7
TS2	459	18.0	3.8	0.48	0.41	0.13	3
**Mean (±SD)**	**720 (±667.3)**	**13.9 (±12.8)**	**3.7 (±0.21)**	**0.48 (±0.033)**	**0.47 (±0.043)**	**0.015 (±0.089)**	**2.6 (±2.0)**
**overall mean (±SD)**	**1003 (±1115.8)**	**12.5 (±9.9)**	**3.7 (±0.27)**	**0.47 (±0.031)**	**0.44 (±0.046)**	**0.050 (±0.087)**	**2.1 (±1.7)**

%F: percentage of female plants in the population; A: the mean number of alleles per population; H_E_: the expected heterozygosity; H_O_: the observed heterozygosity; F_IS_: the approximated inbreeding coefficient,LD: number of allele pairs for which linkage disequilibrium occurred and SD: standard deviation.

**Table 2:** Parameter estimates of the final GLM analyses after model reduction (n = 27).

	Model	age		pop size		%F		pop size*%F	
	R^2^	F	β	F	β	F	β	F	β
A	0.15	-	-	6.4	0.35*	6.3	-0.92*	7.3	0.16*
H_O_	0.27	10.7	-0.025**	-	-	-	-	-	-
F_IS_	0.13	4.8	0.068*	-	-	-	-	-	-
LD	0.28	-	-	10.9	-0.87**	-	-	-	-

A: mean number of alleles per population; H_O_: observed heterozygosity; F_IS_: the approximated inbreeding coefficient, LD: number of allele pairs for which linkage disequilibrium occurred and %F: percentage of female plants in the population. Significance: * 0.05≥ *P*-value > 0.01 ** 0.01≥*P*-value > 0.001.

### Genetic differentiation

**Figure 4:**
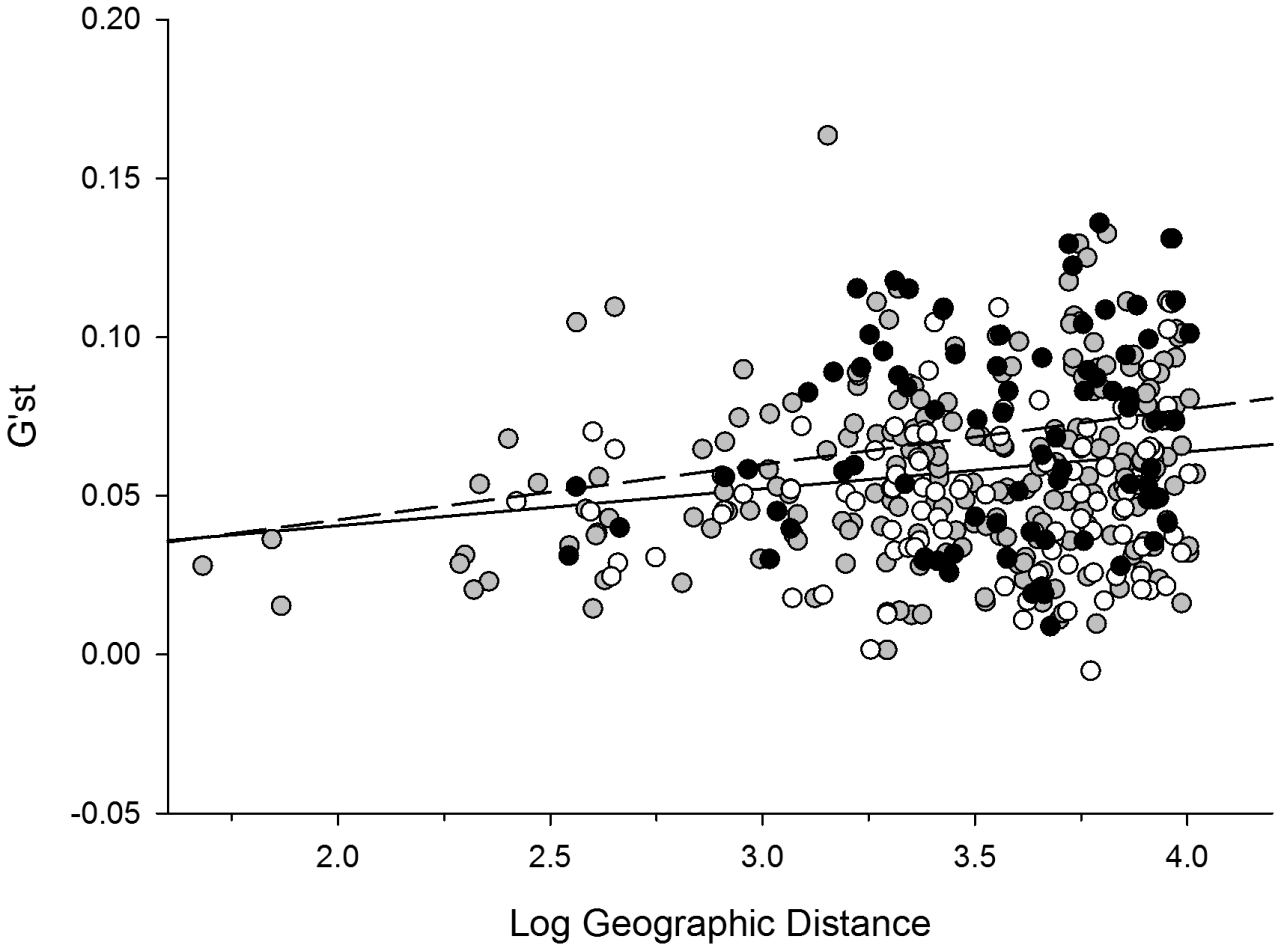
Isolation by distance graph. Correlation between Pairwise G’_ST_ values and logarithmic transformed geographic distance for all populations (all circles, continuous line (R_M_ = 0.17; *β* = 0.012; *P* = 0.001)), old populations (black circle, dashed line (R_M_ = 0.19; *β* = 0.018; *P* = 0.043)) and recent populations (open circle, no regression line (R_M_ = 0.069; *β* = 0.0044; *P* = 0.25)).

The overall genetic differentiation among all populations was low (F_ST_ = 0.040; G’_ST_ = 0.058; Jost’s D = 0.039), but significant according to the AMOVA based on F_ST_ (*P*<0.001) (Tables S1 & S2 in supporting information). Genetic differentiation based on F_ST_, G’_ST_ and Jost’s D was significantly higher for old populations than for recent populations (Table 3). The average geographic distance separating populations, however, was not significantly different between old and recent populations (Table 3). The AMOVA results indicated that no significant overall genetic differentiation occurred between recent and old populations (F_RT_ <0.0001, *P* = 0.55). We observed significant isolation by distance based on F_ST_, G’_ST_ and Jost’s D when including all populations, a trend that was even stronger when only including old populations. However, no isolation by distance was observed when including only recent populations (Fig. 4).

**Table 3:** Parameter estimates of performed bootstrapping analysis on pairwise differentiation for recent and old populations and differences between recent and old populations tested based on bootstrap t-tests.

	recent pop.		old pop.		t-test
	mean	CI	mean	CI	mean difference
FST	0.030	0.029–0.032	0.035	0.033–0.037	–0.0048**
G'ST	0.048	0.043–0.053	0.069	0.062–0.076	–0.022***
Jost's D	0.031	0.028–0.035	0.047	0.042–0.052	–0.016***
Geo. Dist. (km)	4.26	3.71–4.83	4.42	3.84–5.01	–0.16

All tests are based on 9999 bootstraps. CI: 95% confidence intervals. Significance: ** 0.01≥ *P*-value > 0.001 *** 0.001≥ *P*-value.

### Overall genetic structure

**Figure 5:**
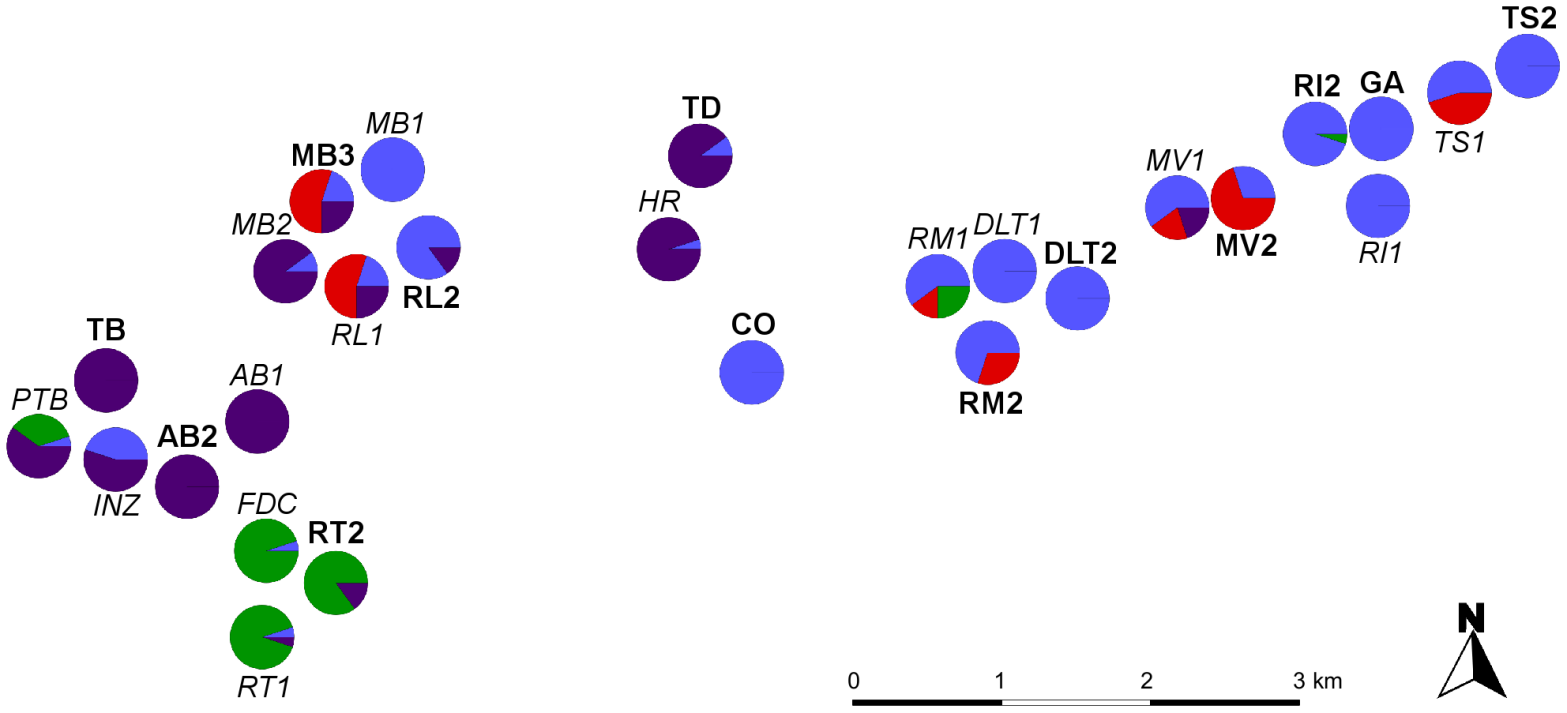
Map of the sampled recent populations (italics) and old populations (bold) of *Origanum vulgare*. Pie slices correspond to population membership to the four genetic groups defined by the Bayesian assignment analysis of Structure [55]. Group 1: blue, group 2: red, group 3: green, group 4: purple. Population codes correspond to those in table 1.

No significant clustering of the sampled populations was found using the admixture model in STRUCTURE. Individuals from the different populations were randomly assigned to one of the inferred groups (1 to K) at all values of *K* between 2 and 16. Using the admixture model with the LOCPRIOR option we observed a true value of 4 for *K*, as indicated by a maximum value of Δ*K* at this value. We observed a geographical clustering of three of these four genetic groups, with group 1 mainly restricted to populations in the east of the study area, and groups 3 and 4 mainly restricted to the west of the study area (Fig. 5, Table S3).

**Figure 6:**
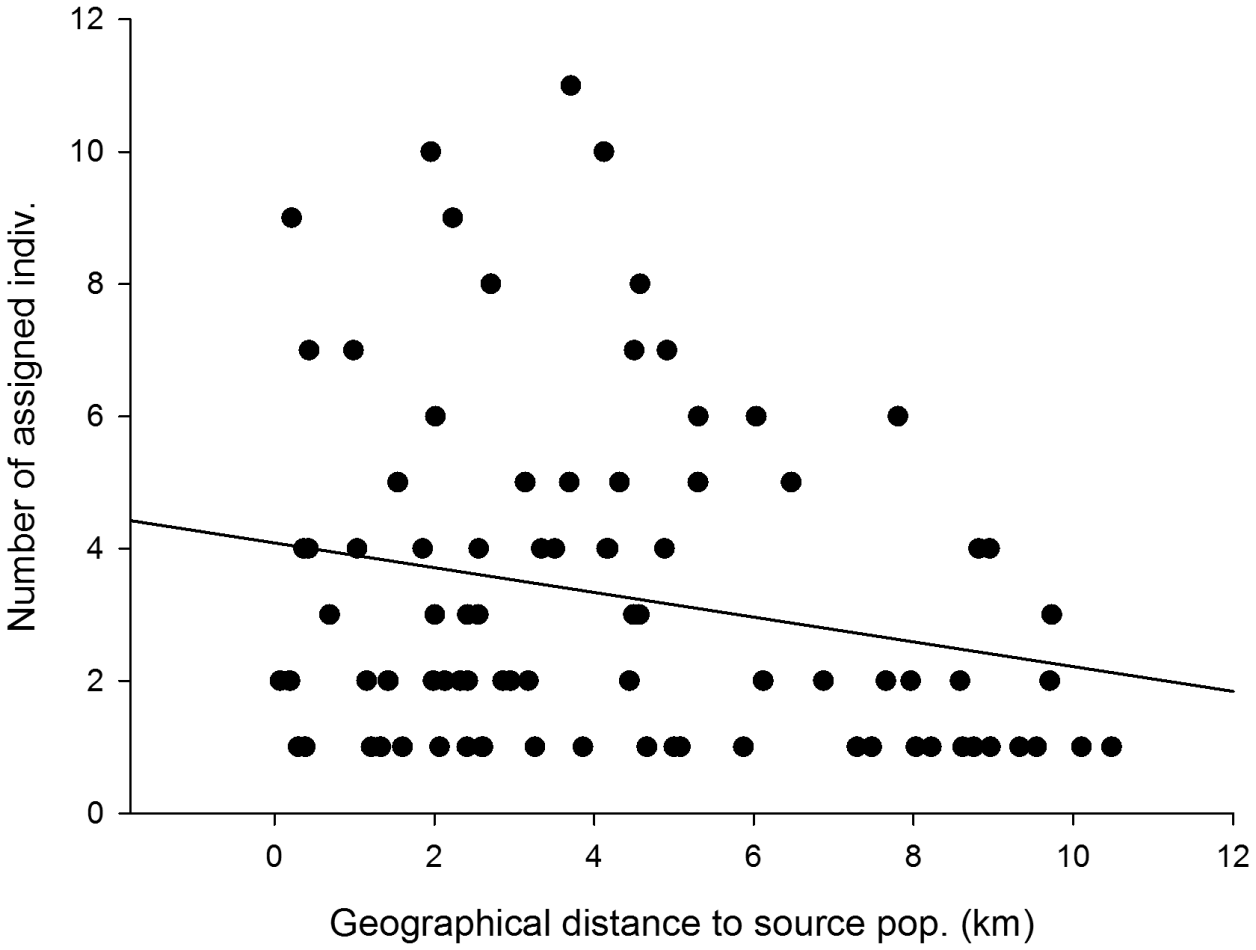
Isolation effect on genetic assignment. Correlation between the number of individuals of recent populations assigned to a source (old) population and the distance of the recent population to this source population (*β* = –0.25, *P* = 0.018). Data points represent assignment data from all 14 recent populations (Table 4).

Plants of individual recent populations were assigned to a mean of 6.1 old populations based on Geneclass2, indicating high gene flow between the different populations (Table 4). Individuals were more frequently assigned to nearby old populations than to more distant ones, as demonstrated by the significant negative correlation between the geographical distance between each recent population and the different source populations on the one hand, and the number of assigned plant individuals to each of these source populations on the other hand (*β* = –0.25, *P* = 0.018) (Fig. 6). We found a mean value of 0.22 for *φ_p_* over all populations (Table 4).

**Table 4:** Assignment of individuals of recent populations (columns) to old populations (rows).

source ↓	AB1	DLT1	FDC	HR	INZ	MB1	MB2	MV1	PTB	RI1	RL1	RM1	RT1	TS1
AB2	4			1			1	2	1	1	2	1	2	4
CO	1	10	11	4	3	5	2	4	4	7	2	5	5	5
DLT2			1			1								
GA	1	2	1		1		2		2				2	
MB3				2	2	2	2	2	1		7	2		2
MV2	1							1						
RI2					1		1	1					1	
RL2	1												1	
RM2							1							
RT2			1				1				2			
TB	7	5	2	10	9	9	2	6	4	4	3	6	4	3
TD	4	3	4	3	3	3	8	4	7	8	4	6	5	6
TS2	1				1				1					
***φ_p_***	**0.17**	**0.31**	**0.32**	**0.29**	**0.23**	**0.26**	**0.17**	**0.15**	**0.18**	**0.29**	**0.17**	**0.22**	**0.15**	**0.18**

## Discussion

The restoration of large patches of calcareous grassland following tree removal led to fast colonization of *Origanum vulgare*, resulting in several new populations in less than 10 years time. Contrary to our initial predictions, the percentage of female plants was not related to population age or population size, suggesting that no sampling effects had occurred in the sex structure of the founder populations after colonization [38]. This is in contrast with recently founded populations of the gynodioecious *Beta vulgaris* ssp. *maritima*, which showed a higher variability in sex structure compared to older populations [41]. However, in this study, the increase in the percentage of female plants led to an increase in the number of alleles for large populations. This could possibly be explained by reproductive differences between hermaphroditic and female plants (the ‘female advantage’). Since female plants are obligatory outcrossing, a higher number of female plants would increase outcrossing and thus lead to higher genetic diversity [42]. Linkage disequilibrium was also found to increase with decreasing population size in old populations, suggesting the occurrence of genetic drift in these populations, further confirmed by the occurrence of significant isolation by distance. This concurs with previous research on the effects of genetic drift on linkage disequilibrium, where populations with high spatial isolation and small population size were found to have higher levels of linkage disequilibrium [59], [13], [14].

The individuals of the newly established populations were assigned to on average 6.1 different source populations, suggesting considerable gene flow within our study area. This was confirmed by the lack of genetic differentiation between old and recent populations. Nevertheless, we observed a significant effect of the spatial configuration of the grasslands on gene flow, with gene flow into the recent populations mainly originating from nearby source populations. This was also reflected in the spatial aggregation of three of the four genetic groups defined by STRUCTURE. These results are in accordance with the findings of [60], who observed that colonization of *Salix reinii* on Mount Fuji mainly consisted of seed recruitment of nearby populations supplemented by only limited seed recruitment over long distances. The diverse genetic origin of the individuals of recent populations in our study system (*φ_p_* = 0.22) suggests that it more likely follows the ‘migrant pool’ model according to Slatkin [10], rather than the ‘propagule pool’ model. Significant isolation by distance was observed among old population (where colonization is lacking), but was absent among the recent populations (where colonization was occurring). Thus, we may assume that ‘background’ migration, mainly through pollen flow, is limited compared to seed flow at colonization in our system, also suggesting the occurrence of the ‘migrant pool’ model [21]. This model predicts the absence of strong founder effects with respect to both genetic differentiation and genetic diversity in recent populations. Since the genetic composition of the source populations can have disproportionate effects on the genetic diversity of founder populations, the occurrence of founder effects can be expected to be even less likely for populations of species that exhibit low overall genetic differentiation, as is the case in our study system (F_ST_ = 0.040) [16], [36].

Our results indeed showed that recent populations were not more genetically differentiated from each other than old populations. This is in accordance with previous research on the colonization of plant species that are characterized by high levels of gene flow and low overall genetic differentiation. Erickson et al. [61], for example, observed comparable levels of genetic differentiation among old and new populations of *Myrica cerifera* during range expansion on Hog Island off the coast of Virginia (USA). Similar results during primary succession were found after colonization of glacier forelands by *Geum reptans* [62] and *Saxifraga aizoides* [63] in the European Alps, and by *Vaccinium membranaceum* at Mount St Helens in Washington [36]. Also colonization of recent lava flows by *Antirhea borbonica* on the Piton de la Fournaise volcano on La Réunion [64] and by *Nassauvia lagascae var. lanata* on the Lonquimay volcano in Chile [65] showed similar results. Antrobus & Lack [66] observed equal levels of genetic differentiation among recent and old populations of *Primula veris* during secondary succession in young ‘grassland’ fragments in the Oxford region (UK).

Whereas recent populations of *O. vulgare* were found to be little differentiated, old populations showed a higher degree of genetic differentiation. Furthermore, we observed significant isolation by distance for old populations, but not for recent populations. This suggests that old populations are moving towards a migration-drift equilibrium. These populations likely became smaller and more isolated when the grassland area decreased in the years prior to restoration [43]. This could have led to decreased gene flow and increased genetic drift, in turn increasing genetic differentiation. This difference in the extent of among population gene flow (migration) and gene flow during colonization can be expected when seed flow is high throughout the system, but when seedlings experience high levels of inter- or intraspecific competition in old populations, leading to density-dependent mortality among migrants. Within recent founder populations, this mortality can be reduced or even absent due to lower levels of competition [36]. In this case we can observe high gene flow towards recent populations due to seed flow, even when among population migration, mainly due to pollen flow is limited. In several studies, higher genetic differentiation or significant isolation by distance among old populations compared to recent populations has been attributed to lower gene flow, caused by higher geographical distance [21], [35], [67], historical levels of gene flow [68], or differences in population size and genetic drift [69].

As predicted by the ‘migrant pool’ model of Slatkin [10], no strong founder effects with respect to genetic diversity, and no evidence of recent bottlenecks in any of the recent populations was found. The number of alleles, expected heterozygosity and linkage disequilibrium of recent populations were also not significantly different from those of old populations. This is in accordance with several other studies of colonization in species with high levels of gene flow. Similar levels of genetic diversity for old and recent populations have been observed during both primary [61]–[63], [70], [71] and secondary [66], [7] succession. However, also higher genetic diversity in recent populations, compared to old populations has been observed in some instances [35], [36]. These authors argued that this difference can be caused by high rates of population growth after the occurrence of only a weak founder effect at colonization, since the population growth rate is known to affect the severity of genetic founder effects or bottlenecks [11], [35], [72]. Since many of the recent populations in our study system have become relatively large (mean population size of 1267 plants) within a short time period (<10 years), this can also explain the absence of strong founder effects.

Observed heterozygosity, on the other hand, was significantly lower in recent populations of *O. vulgare*, resulting in a higher inbreeding coefficient in these populations. The mean F_IS_ value of 0.083 for recent populations was low however, and together with the absence of fitness effects on germination rate or seed weight in these recent populations, this suggests that inbreeding depression is absent. We can expect that future gene flow and population expansion of the recent populations will probably lead to a decreasing F_IS_ [72].

Finally, the absence of pronounced founder effects in this study may also partly be explained by the occurrence of seed dispersal through time [71]. *Origanum vulgare* is known to form a persistent seed bank in our study area [73]. All restored grasslands were historically grasslands before their afforestation and degradation. Therefore, it is not impossible that some seeds of *O. vulgare* were still present within the soil seed bank at the time of restoration. Recruitment from these seeds could have influenced the observed levels of gene flow, cf. [14]. In this sense, gene flow towards the recent founder populations can be seen as the sum of temporal gene flow through the germination of dormant seeds, and spatial gene flow. Additionally, as stated before, pollen flow may also have occurred between the old and recent populations. Further analyses based on the assignment of seeds and seedlings to parent plant should be used to estimate the relative rates of pollen- and seed flow in our study system [74].

This study demonstrated that spontaneous plant colonization after habitat restoration can lead to new and viable populations, overcoming potentially important genetic founder effects, when several source populations are nearby. It has been suggested that the severity of founder effects is largely dependent upon the characteristics of the study area and the traits of the focal species, through mediating gene flow and population growth rate after colonization [22], [35], [20], [72]. This likely explains the absence of any founder effects in many studies. The rapid buildup of genetic diversity in restored populations, combined with low among population differentiation, can be expected to contribute positively to the overall viability of the *O. vulgare* metapopulation and also to mitigate the consequences of the genetic drift observed in the original source populations.

## Supporting Information

Table S1 **Pairwise genetic differentiation among** ***Origanum vulgare*** **populations.** Lower left triange: F_ST_ estimates; Upper right triange: G’_ST_ estimates. Significant values are shaded in grey.
(XLS)

Table S2 **Pairwise genetic differentiation among** ***Origanum vulgare*** **populations.** Lower left triangle: Jost’s D estimates Significant values are shaded in grey.
(XLS)

Table S3 **Inferred ancestry of individual plants.** Analysis based on the Bayesian clustering of STRUCTURE 2.3.3 for K = 4, using the admixture with correlated alleles model with LOCPIOR option. q_g_ =  the probability of individual ancestry from group g. Each individual was assigned to the group with the highest q_g_ value (assigned group).
(XLS)
